# QSOX1 Inhibits Autophagic Flux in Breast Cancer Cells

**DOI:** 10.1371/journal.pone.0086641

**Published:** 2014-01-24

**Authors:** Laura Poillet, Nicolas Pernodet, Michaël Boyer-Guittaut, Pascale Adami, Christophe Borg, Michèle Jouvenot, Régis Delage-Mourroux, Gilles Despouy

**Affiliations:** 1 Université de Franche-Comté, Estrogènes, Expression Génique et Pathologies du Système Nerveux Central, U.F.R. Sciences et Techniques, Besançon, Doubs, France; 2 Université de Franche-Comté, Inserm UMR 1098, Relation Hôte Greffon et Ingénierie Cellulaire et Génique, Besançon, Doubs, France; Sun Yat-sen University Medical School, China

## Abstract

The QSOX1 protein (Quiescin Sulfhydryl oxidase 1) catalyzes the formation of disulfide bonds and is involved in the folding and stability of proteins. More recently, QSOX1 has been associated with tumorigenesis and protection against cellular stress. It has been demonstrated in our laboratory that QSOX1 reduces proliferation, migration and invasion of breast cancer cells *in vitro* and reduces tumor growth *in vivo*. In addition, QSOX1 expression has been shown to be induced by oxidative or ER stress and to prevent cell death linked to these stressors. Given the function of QSOX1 in these two processes, which have been previously linked to autophagy, we wondered whether QSOX1 might be regulated by autophagy inducers and play a role in this catabolic process. To answer this question, we used *in vitro* models of breast cancer cells in which QSOX1 was overexpressed (MCF-7) or extinguished (MDA-MB-231). We first showed that QSOX1 expression is induced following amino acid starvation and maintains cellular homeostasis. Our results also indicated that QSOX1 inhibits autophagy through the inhibition of autophagosome/lysosome fusion. Moreover, we demonstrated that inhibitors of autophagy mimic the effect of QSOX1 on cell invasion, suggesting that its role in this process is linked to the autophagy pathway. Previously published data demonstrated that extinction of QSOX1 promotes tumor growth in NOG mice. In this study, we further demonstrated that QSOX1 null tumors present lower levels of the p62 protein. Altogether, our results demonstrate for the first time a role of QSOX1 in autophagy in breast cancer cells and tumors.

## Introduction

Quiescin Sulfhydryl Oxidase 1 (QSOX1) was described in our laboratory as an estrogen-regulated gene in guinea-pig endometrial glandular epithelial cells [1]. The human QSOX1 gene, localized on chromosome 1 (1q24), encodes two major protein isoforms, QSOX1-S (66 kDa) and QSOX1-L (82 kDa), resulting from an alternative mRNA splicing process (QSOX1-S: NM_001004128; QSOX1-L: NM_002826) [2], [3]. Immunoelectron microscopy experiments have shown that the QSOX1 protein is linked to the endoplasmic reticulum (ER) membrane, the Golgi apparatus and to secretory granules [4] and has also been detected in culture supernatants and extracellular spaces [1], [5], suggesting its extracellular secretion. QSOX1 proteins belong to the flavin adenine dinucleotide (FAD)-dependent sulfhydryl oxidase family and catalyze the formation of disulfide bonds in unfolded proteins [6]. This activity has been proposed to play an important role for incorporation of laminin in extracellular matrix (ECM) synthesized by fibroblasts and the adhesion of cancer cells to the ECM [7].

More recently, QSOX1 has been associated to cancer and protection against cellular stress. In fact, several studies have shown a deregulation of QSOX1 expression in cancer cells [8], [9], [10] and its involvement in tumorigenesis. Indeed, in our laboratory, we have demonstrated that QSOX1 reduces proliferation, migration, invasion *in vitro* and tumorigenesis *in vivo* which is in agreement with our findings, indicating that a high QSOX1 expression is associated with a better survival for breast invasive ductal carcinomas patients [11]. These results are in agreement with those previously obtained *in vitro* regarding the role of QSOX1 in proliferation and cell adhesion [1], [12], [13], [14], [15]. On the contrary, it has been demonstrated that QSOX1 promotes invasion and proliferation of pancreatic and breast tumor cells *in vitro* and that QSOX1 mRNA is a predictive marker of poor survival in luminal B tumor [16], [17]. Recently, Soloviev and colleagues have demonstrated that QSOX1 mRNA is overexpressed in breast ductal carcinoma and that this increase is correlated to the tumor grade [18]. Therefore, it is now clear that the role of QSOX1 in cancer is complex mainly because of the existence of its different transcripts and that its function seems to depend on the stage and type of tumor.

Furthermore, we have shown that QSOX1 protects cells against cellular stressors. Indeed, QSOX1 mRNA and protein levels are increased following an oxidative or an ER stress and QSOX1 protects against stress-induced-cell death [15] (unpublished data).

Cancer and protection against cellular stressors are two processes that have previously been linked to autophagy. Autophagy, a cellular degradation process involved in the degradation and recycling of damaged proteins, organelles and other cytoplasmic constituents, occurs at low basal levels in almost every cell type to maintain cellular homeostasis. Following a metabolic stress, such as nutrient starvation, oxidative stress or ER stress, autophagy is induced to provide nutrients and energy allowing cell survival [19]. Three types of autophagy have been described: macroautophagy, microautophagy and chaperone-mediated autophagy [20]. Macroautophagy (hereafter called autophagy) is a multi-step process involving initiation, elongation, maturation and degradation steps. The initiation step is characterized by the induction of a unique double membrane structure called the phagophore that sequesters part of the cytoplasm, soluble proteins and/or organelles. The elongation and closure of this phagophore results in the formation of a double-membrane organelle called the autophagosome which ultimately fuses with the lysosome to form the autophagolysosome, leading to the degradation of its contents [21].

Besides its role in cellular homeostasis, autophagy has been described to be involved in various cancers such as breast cancer [22], [23], [24]. However, the role of autophagy in cancer formation and growth is complex and context-dependent. During the early stages of tumorigenesis, autophagy acts as a tumor suppressor mechanism by preventing cytoplasmic damage, genomic instability and inflammation which usually lead to cancer initiation and development [25], [26]. Moreover, expression levels of proteins involved in autophagy are reduced or lost in several types of cancers [27], [28], [29]. For example, *Beclin1* gene is deleted in 50% of breast cancer [27], [30]. On the contrary, during the later stages of cancer growth, autophagy promotes survival of cancer cells under conditions of metabolic stress. Indeed, the microenvironment of cancer cells presents reduced levels of nutrients, oxygen and growth factors leading to decreased oxidative phosphorylation, decreased ATP production and limited cancer cell proliferation [31], [32]. For example, it has been shown that the inhibition of autophagy by a *fip200* (FAK family-interacting protein of 200 kDa) gene deletion in a mouse model of human breast cancer leads to reduced tumor initiation and progression by both the impairment of tumor cell proliferation and the induction of immune surveillance [33].

Given the function of QSOX1 in cancer and protection against cellular stressors, two processes linked to autophagy, the purpose of our study was to determine whether QSOX1 could be regulated by autophagy inducers and might play a role in this catabolic process that could explain its function during breast tumor development. We showed in this study, that QSOX1 expression is increased following nutrient stress-induced autophagy and maintains cellular homeostasis. Our results also indicated that QSOX1 inhibits autophagy through the inhibition of autophagosome/lysosome fusion in breast cancer cell line. Therefore, we propose that its inhibitory effect on autophagy might explain its function in cancer cell invasion and tumor growth.

## Materials and Methods

### Reagents and Antibodies

Cell culture reagents were purchased from Invitrogen. Earle’s balanced salt solution (EBSS, E3024), bafilomycin A1 (B1793), 3-methyladenine (3-MA) (M9281) and wortmannin (W1628) were purchased from Sigma Aldrich. For the western blotting experiments, the following antibodies were used: polyclonal anti-rat QSOX1 [5] diluted at 1∶7500, polyclonal anti-LC3 (Sigma, L8918) diluted at 1∶3000, monoclonal anti-p62 (Santa Cruz, sc-28359) diluted at 1∶1500, polyclonal anti-actin (Sigma, A5060) diluted at 1∶15000, polyclonal anti-rabbit (P.A.R.I.S, BI2407) diluted at 1∶10000 and polyclonal anti-mouse (P.A.R.I.S, BI24130) diluted at 1∶10000. For the immunofluorescence experiments, the following antibodies were used: monoclonal anti-p62 antibody (Santa Cruz, sc-28359) diluted at 1∶250, monoclonal anti-mouse lysosomal-associated membrane protein 1 (LAMP1) (Abcam, Ab25630) diluted at 1∶100, Alexa Fluor 555 goat anti-mouse (Life technologies, A-21422) diluted at 1∶800.

### Cell Culture and Treatment

Two breast cancer cell lines were used: MCF-7 in which QSOX1-S is overexpressed (MCF-7 QSOX1S-1 and QSOX1S-2) and MDA-MB-231 in which QSOX1 is extinguished (MDA-MB-231 shQSOX1-1 and shQSOX1-2). In every experience, we compared these stable cell lines to the control ones (MCF-7 C transfected with an empty control vector and MDA-MB-231 shC expressing a control shRNA). These cellular models have previously been described [11]. MCF-7 QSOX1S-1 and QSOX1S-2 cells show similar overexpression of QSOX1 whereas MDA-MB-231 shQSOX1-1 and shQSOX1-2 cells show 55% and 92% of QSOX1 extinction, respectively [11]. MCF-7 cells were chosen to overexpress QSOX1 since this protein is weakly expressed in these cells. On the contrary, MDA-MB-231 cells were selected because they present a high expression of endogenous QSOX1 [11], [14].

Cells were cultured in Dulbecco’s minimum essential medium (DMEM) (PAA, E15-891) supplemented with 100 µg/ml penicillin/streptomycin (PAA, P11-010) and 5% fetal bovine serum (FBS) (PAA, A15-101) in a 5% CO2 incubator at 37°C. To inhibit autophagosome/lysosome fusion, cells were incubated for 8 h in complete medium supplemented with 100 nM bafilomycin A1. To induce autophagy, cells were incubated in EBSS for 2 to 8 h at 37°C. To inhibit autophagy, cells were incubated in complete medium supplemented with 10 mM 3-MA or with 100 nM wortmannin for 24 h.

### Western Blotting

Cells were scraped, harvested and lysed in a RIPA buffer (50 mM Tris-HCl, pH 8, 150 mM NaCl, 1% Triton X100, 0.5% DOCA, 0.1% SDS) supplemented with 0.1% protease inhibitors (104 mM AEBSF, 1.5 mM pepstatin A, 1.4 mM E-64, 4 mM bestatin, 2 mM leupeptin, 80 µM aprotinin). Protein lysates were sonicated for 5 s before loading (Sonics and Materials), separated on a 10–12.5% sodium dodecyl sulfate-polyacrylamide gel electrophoresis (SDS-PAGE) before being transferred onto a polyvinylidene difluoride (PVDF) membrane (Bio-Rad, 162–0177). The membrane was blocked with 5% nonfat milk in Tris-buffered saline with Tween 20 (TBS-T) (20 mM Tris-HCl, pH 7.6, 137 mM NaCl, 0.1% Tween 20) and incubated with primary antibodies at the previously indicated dilutions. Immunoreactive bands were detected using goat horseradish peroxidase (HRP)-coupled secondary anti-mouse or anti-rabbit antibodies and the *p*-coumaric acid- enhanced chemiluminescent (PCA-ECL) solution [34].

### Cell Viability

For the MTT assay, cells (1.3 × 10^4^ cells/well) were cultured in a 96-well plate and incubated with EBSS for 4 and 8 h. After the removal of the supernatant, 100 µl of 100 mM MTT solution (Sigma, M2128) in Hank’s were added to the cells. After a 2 h incubation, the formazan crystals were dissolved in dimethyl sulfoxide (DMSO) (50 µl) and the absorbance was quantified at 549 nm using a microplate reader (Multiskan FC, ThermoScientific). All experiments were performed in 8 replicates, and the relative cell viability (%) was normalized to the untreated control cell.

For the trypan blue exclusion assay, cells (7.6×10^4^ cells/well) were seeded in a 24-well plate in duplicate and incubated with EBSS for 4 and 8 h. Cells were then collected by trypsinization, stained with trypan blue (0.04%) (Sigma, T8154) and cells were counted in triplicate. Data were obtained from two independent experiments.

### Immunofluorescence and Confocal Microscopy

The green fluorescent protein-microtubule-associated protein light chain 3 (GFP-LC3) plasmid was kindly provided by Dr. Elazar (Weizmann Institute, Israël). For transient GFP-LC3 transfection, MCF-7 and MDA-MB-231 cells were plated on coverslips in 6-well plates on coverslips at a density of 4.5×10^5^ and 3×10^5^ cells/well, respectively. Plasmids were transfected using the Jetprime reagent (Polyplus transfection, 114-07) according to the manufacturer’s protocol. After the designated treatments, cells were washed with phosphate-buffered saline (PBS) and fixed with 4% paraformaldehyde (PFA) (Sigma, P6148) in PBS for 15 min at room temperature. The cells were then examined and photographed using a confocal microscope (Olympus Fluoview FV1000).

For Lysotracker staining, cells were incubated for 1 h in complete medium supplemented with 500 nM Lysotracker red DND-99 (Invitrogen, L75-28). Cells were then washed with PBS and fixed with 4% PFA in PBS for 15 min at room temperature. Cells were then analyzed by confocal microscopy.

For LAMP1 immunofluorescence, cells were washed with PBS and fixed with 4% PFA in PBS for 15 min at room temperature. Cells were then permeabilized with 0.2% Triton-X100 in PBS for 5 min, washed with PBS, blocked with 5% bovine serum albumin (BSA) (Sigma, A6793) in PBS for 30 min, incubated with an anti-mouse LAMP1 primary antibody overnight at 4°C and finally with an Alexa Fluor 555 goat anti-mouse for 1 h at the previously indicated dilutions. The cells were analyzed using a confocal microscope. Each picture is representative of a typical cell staining observed in 10 fields chosen at random.

GFP-LC3 and Lysotracker red or LAMP1 colocalization was analyzed using the ImageJ software and the Pearson’s coefficient. For each cell line, 35 cells were randomly selected.

For immunofluorescence staining of tumor tissue sections, the slides were incubated at 95°C for 40 min in sodium citrate buffer (10 mM sodium citrate, pH 6). The tissue sections were then incubated overnight with the previously described p62 antibody and incubated for 1 h at room temperature with an Alexa Fluor 555 goat anti-mouse secondary antibody at previously indicated dilutions and with DAPI (1∶333) (AAT bioquest, 17510) in PBS for 10 min. After each incubation, the slides were rinsed thrice in 1% PBS-Triton X-100. After being mounted in PBS-glycerol mounting medium, the slides were observed and analyzed using confocal microscopy.

### Real-time PCR

Total RNAs were extracted as previously described [15]. For real time RT-PCR analysis, 2 µg of total RNAs were reverse transcribed using the RevertAid M-MulV Reverse Transcriptase (Fermentas, EP0441) according to the manufacturer’s protocol.

Quantitative PCR was performed using the Step One Real Time PCR System (Applied Biosystems) and the SYBER Green PCR Master Mix (Applied Biosystems, 4309155). The primers used for QSOX1 (Targeting endogenous QSOX1-S and QSOX1-L mRNA) were p85r: 5′-AATCAAGCATGTGTAAGGCAC-3′ and p85s: 5′-GACCTGACGAGTTGGT-3′ and the ones used for p62 were p62_F_Hs: 5′-ATCGGAGGATCCGAGTGT-3′ and p62_R_Hs: 5′-TGGCTGTGAGCTGCTCTT-3′. The PCR signals were normalized with the endogenous control (H3.3 like histone H3B-2) amplified with the primers His-I: 5′-GCTAGCTGGATGTCTTTTGG-3′ and His-N: 5′-GTGGTAAAGCACCCAGGAA-3′.

### Cell Invasion Assay

50 µl of extra cellular matrix (ECM) gel (1 mg/ml) (Sigma, E6909) were added to the upper chamber and incubated for 5 h at 37°C. 10^5^ cells were subsequently diluted in 250 µl serum-free medium, added to the upper chamber and incubated for 24 h at 37°C in the presence or absence of an autophagy inhibitor (3-MA or wortmannin). The cells on the upper surface were removed using a cotton bud while the remaining invasive cells were fixed with 100% ethanol, stained with 2% crystal violet and images from each membrane were taken. Finally, the invasive cells located in the lower chamber were counted manually in 10 fields of view (FOV).

### Xenograft Experiments

CIEA NOG mice were obtained from Taconic (Germantown, NY, USA) and maintained in the UMR1098 animal facility (agreement number #C25-056-7). Approval for animal experimentation and care was received from the Services Vétérinaires de la Santé et de la Protection Animale delivered by the Ministère de l’Agriculture, Paris, France and experimental procedures were approved by a local ethic committee (Comité d’Ethique Bisontin d’Expérimentation Animale [CEBEA]).

As previously described [11], MDA-MB-231 shC, shQSOX1-1 and shQSOX1-2 cells were subcutaneously inoculated in NOG mice and tumor growth was monitored twice a week. When tumors reached a diameter of 1 cm, mice were sacrificed and each tumor was fixed in formol and photographed.

### Statistical Analysis

Statistical analyses were carried out using a Student’s t test. A p value <0.05 was considered statistically significant.

## Results

### QSOX1 is Induced Following Nutrient Stress and Protects against Cell Death

**Figure 1:**
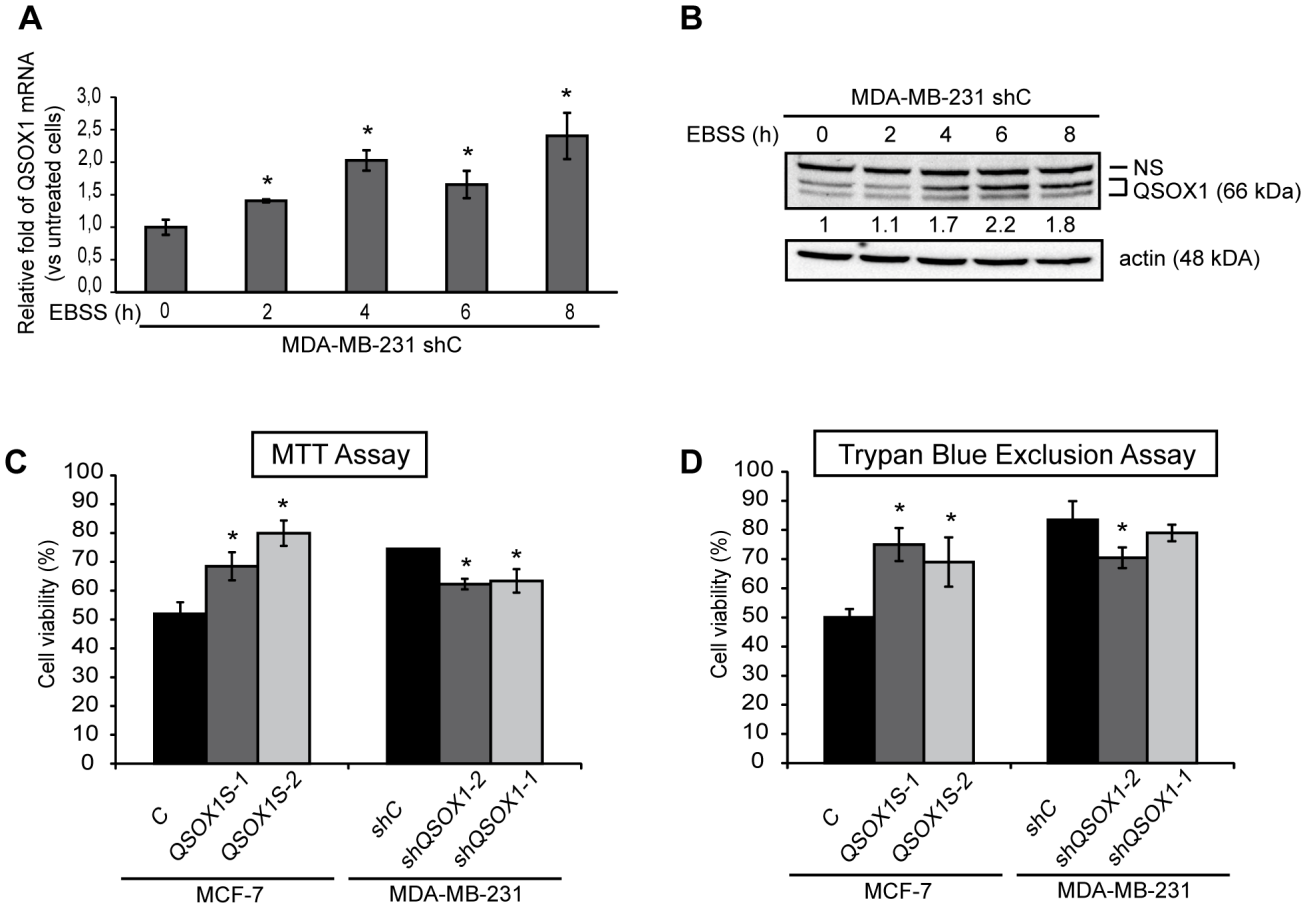
QSOX1 is induced following a nutrient stress and protects against cell death. (**A, B**) MDA-MB-231 shC cells were cultured in the presence or absence of EBSS for 2, 4, 6 and 8 h. (**A**) After a reverse transcription step, relative QSOX1 mRNA expression was determined by qPCR. H3B-2 mRNA was used for normalization. Data are means ± S.D. of two independent experiments performed in triplicate. *P<0.05, compared to the control. (**B**) Cells were lysed and total proteins (50 µg) were separated on a 10% SDS-PAGE followed by immunoblotting using anti-QSOX1 and anti-actin antibodies. QSOX1 levels were quantified using the Image Lab software. NS: Non specific signal. (**C, D**) MCF-7 C, QSOX1S-1, QSOX1S-2 and MDA-MB-231 shC, shQSOX1-1, shQSOX1-2 cells were cultured in the presence or absence of EBSS for 8 h. Cell viability was estimated using a MTT assay (**C**) or a trypan blue exclusion assay (**D**). Results were expressed as a ratio between treated and untreated cells. Data are means ± S.D. of two independent experiments performed in 8 replicates for the MTT assay and in duplicate for the trypan blue exclusion assay). *P<0.05 compared to the control.

Our laboratory has previously described that oxidative [15] or ER stress (unpublished data) induces QSOX1 expression and that this protein protects cells against these cellular stresses. Since these stressors can also induce autophagy, we investigated whether amino acid starvation could induce QSOX1 mRNA and protein expression. To do so, starvation was induced in MDA-MB-231 shC cells by an EBSS treatment of 2, 4, 6 or 8 h. An increase in QSOX1 expression was detected from 2 h of EBSS treatment. The highest fold increase was observed after 8 h of EBSS treatment for QSOX1 mRNA expression (2.5) ***(*** ***Figure 1A*** ***)*** and after 6 h of EBSS treatment for QSOX1 protein expression (2.2) ***(*** ***Figure 1B*** ***)***. These results demonstrate that nutrient stress can induce QSOX1 mRNA and protein.

Since amino acid starvation can regulate cell viability, we then examined whether QSOX1 could protect the cells against such stress. To do so, we used two breast cancer cell lines: MCF-7 in which QSOX1-S is overexpressed (MCF-7 QSOX1S-1 and QSOX1S-2) and MDA-MB-231 in which QSOX1 is extinguished (MDA-MB-231 shQSOX1-1 and shQSOX1-2) [11]. Overexpression was induced in MCF-7 because of their initial low expression of QSOX1 whereas extinction was performed in MDA-MB-231 cells because of their endogenous high expression of QSOX1. The viability of these cellular models was assessed using a MTT assay following an 8 h treatment with EBSS. MCF-7 QSOX1S-1 and QSOX1S-2 cells (65% and 76%, respectively) presented a higher viability than MCF-7 C cells (52%). On the other hand, MDA-MB-231 shQSOX1-2 and shQSOX1-1 cells (62% and 63%, respectively) showed a lower viability compared to MDA-MB-231 shC cells (74%) ***(*** ***Figure 1C*** ***).***

The percentage of living cells was also determined using a trypan blue exclusion test. The results were similar to those obtained with the MTT assay. MCF-7 QSOX1S-1 and QSOX1S-2 cells (75% and 69%, respectively) showed a higher viability than MCF-7 C cells (50%), while MDA-MB-231 shQSOX1-2 and shQSOX1-1 cells (73% and 81%, respectively) presented a lower viability compared to MDA-MB-231 shC cells (88%) ***(*** ***Figure 1D*** ***).*** Similar results were also obtained after a 4 h treatment with EBSS (Data not shown). Altogether, our data demonstrate that QSOX1 is induced following amino acid starvation and protects against a nutrient stress-induced cell death.

### QSOX1 Inhibits Autophagic Flux

During autophagy, the microtubule-associated-protein light chain 3 (LC3) is cleaved to give the cytoplasmic mature form (LC3-I). During autophagosome elongation, LC3-I is conjugated to phospholipids to give the membrane-associated form (LC3-II). Thus, the amount of LC3-II is directly correlated with the number of autophagosomes and is considered as an autophagosome marker. The protein p62/SQTM1, which is specifically degraded during autophagy, has also been described as a marker of the autophagic flux.

**Figure 2:**
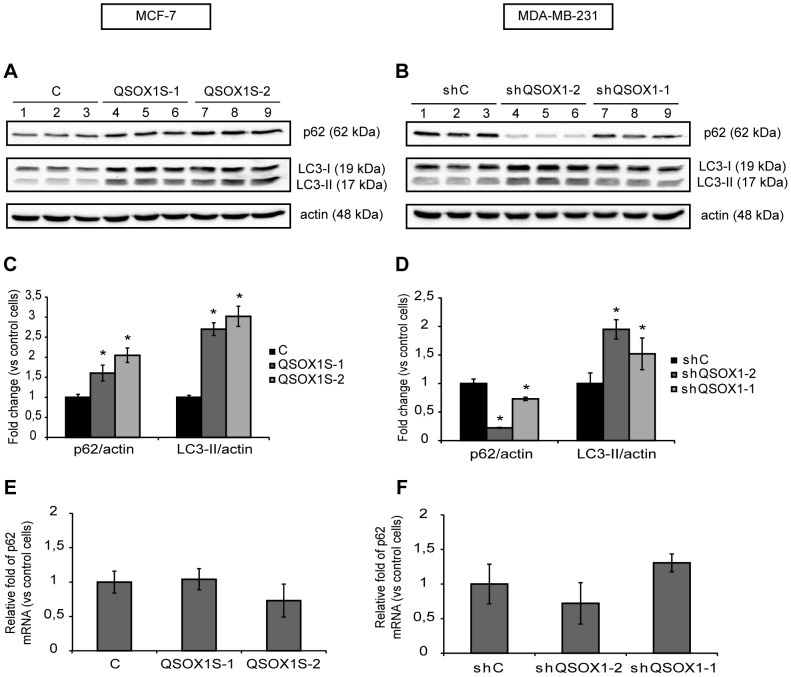
QSOX1 regulates the levels of autophagic markers, p62 and LC3-II. (**A**) MCF-7 C (lanes 1–3), QSOX1S-1 (lanes 4–6), QSOX1S-2 (lanes 7–9) and (**B**) MDA-MB-231 shC (lanes 1–3), shQSOX1-2 (lanes 4–6), shQSOX1-1 (lanes 7–9) cells were cultured for 24 h. Cells were then lysed and total proteins (40 µg) were separated on a 12.5% SDS-PAGE followed by immunoblotting using anti-p62, anti-LC3 and anti-actin antibodies. (**C and D**) p62 and LC3-II levels, observed on the western blot in A and B respectively, were quantified using the Image Lab software. Data are means ± S.D. of one representative experiment performed in triplicate. *P<0.05 compared to the control. (**E and F**) After reverse transcription, relative p62 mRNA levels in MCF-7 C, QSOX1S-1, QSOX1S-2 (E) and MDA-MB-231 shC, shQSOX1-1, shQSOX1-2 (F) cells were determined by qPCR. H3B-2 mRNA was used for normalization. Data are means ± S.D. of one representative experiment performed in triplicate.

We first investigated whether QSOX1 could regulate autophagy by studying the changes in p62 and LC3-II protein levels in our models during basal and induced autophagy. As shown in ***Figures 2A and C***, overexpression of QSOX1 in MCF-7 QSOX1S-1 and QSOX1S-2 cells leads to an increase in p62 levels compared to the levels observed in MCF-7 C cells (1.4 and 1.8 fold, respectively). On the contrary, knock-down of QSOX1 in MDA-MB-231 shQSOX1-2 and shQSOX1-1 cells leads to a decrease of p62 levels compared with those observed in MDA-MB-231 shC cells (0.8 and 0.2 fold, respectively) ***(*** ***Figures 2B and D*** ***)***. Moreover, in the MDA-MB-231 shQSOX1-1 and shQSOX1-2 cells, p62 levels could be correlated to QSOX1 mRNA levels in these different cell lines which are decreased of 55% and 92%, respectively [11]. Since it has been shown that p62 transcription can be regulated by different pathways such as the NRF2 pathway during oxidative stress [35], the Ras/MAPK pathway [36] or the JNK/c-Jun pathway [37], we checked whether p62 protein level variations were due to a transcriptional regulation. In our cellular models, no significant changes in p62 mRNA levels were observed ***(*** ***Figures 2E and F*** ***)***, confirming that the change in p62 protein levels was not due to a transcriptional regulation of the p62 gene but could be attributed to its degradation by the autophagic process. These results suggested that QSOX1 inhibits the autophagic flux. Interestingly, LC3-II protein levels were increased in both our knockdown and overexpression models ***(*** ***Figures 2A, B, C, D*** ***)***. Since LC3-II is degraded after autophagolysosome formation, the amount of LC3-II protein is not directly linked to the autophagic flux but rather to the number of autophagosomes at a particular time. An increase in LC3-II protein levels might therefore represent either an increased autophagosome formation or a blockade in autophagosomal maturation and degradation [***38***].

**Figure 3:**
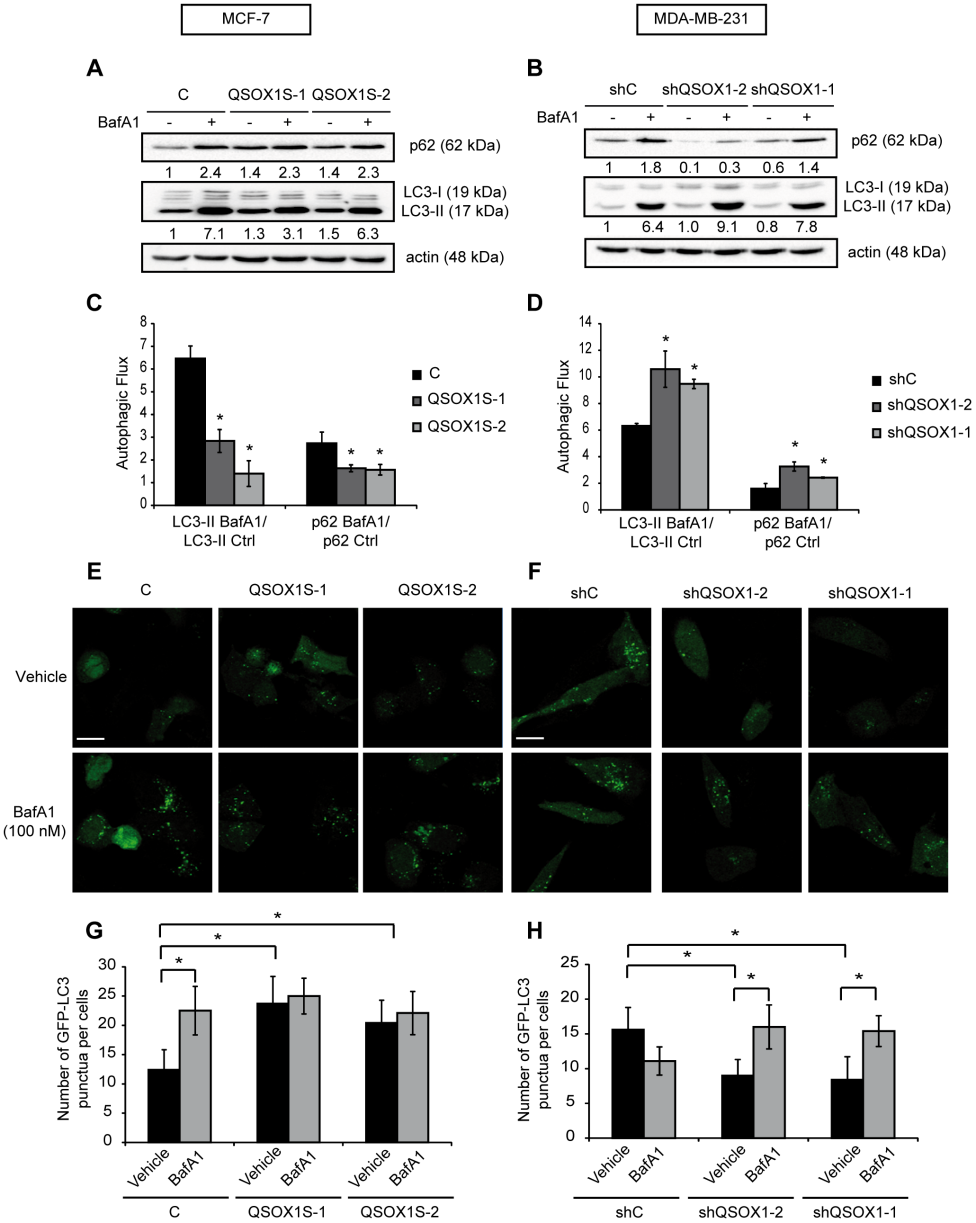
QSOX1 inhibits autophagic flux. (**A**) MCF-7 C, QSOX1S-1, QSOX1S-2 and (**B**) MDA-MB-231 shC, shQSOX1-2, shQSOX1-1 cells were cultured in complete medium with or without 100 nM bafilomycin A1 for 8 h. Cells were then lysed and total proteins (40 µg) were separated on 12.5% SDS-PAGE followed by immunoblotting with anti-p62, anti-LC3 and anti-actin antibodies. p62 and LC3-II levels were quantified using the Image Lab software. (**C and D**) The autophagic flux, observed in A and B respectively, was determined as the ratio of LC3-II protein levels in the presence of bafilomycin A1 versus the levels in the absence of bafilomycin A1. Data are means ± S.D. of three independent experiments. *P<0.05 compared to the control. (**E**) MCF-7 C, QSOX1S-1, QSOX1S-2 and (**F**) MDA-MB-231 shC, shQSOX1-1, shQSOX1-2 cells were transfected with the pGFP-LC3 vector. 24 h after transfection, cells were incubated with or without 100 nM bafilomycin A1 for 8 h. GFP-LC3 puncta were then analyzed by confocal microscopy. Each picture is representative of a typical cell staining observed in 10 fields chosen at random. Scale bar represents 20 µm. (**G and H**) GFP-LC3 puncta, observed in E and F respectively, were counted using the ImageJ software. For each group, 20 cells were randomly selected. Data are means ± S.D. of three independent experiments. *P<0.05 compared to the control.

To study the autophagic flux, we examined the effect of QSOX1 levels variations on autophagic flux by studying p62 and LC3-II levels in the presence or absence of an autophagosome/lysosome fusion inhibitor, bafilomycin A1. In our experiments, bafilomycin A1 led to an increase in p62 and LC3-II levels in MCF-7 C cells whereas it induced a limited effect on p62 and LC3-II levels in MCF-7 QSOX1S-1 and QSOX1S-2 cells ***(*** ***Figure 3A*** ***)***. Similarly, after treatment with bafilomycin A1, MDA-MB-231 shQSOX1-2 and shQSOX1-1 cells presented increased p62 and LC3-II levels whereas MDA-MB-231 shC cells displayed only a slight change in these proteins levels ***(*** ***Figure 3B*** ***)***. These results indicate that bafilomycin A1 has little effect on p62 and LC3-II levels in cells expressing QSOX1.

Moreover, we quantified the ratio of LC3-II levels in the presence of bafilomycin A1 over LC3-II levels in the absence of bafilomycin A1, a ratio that has been previously used to describe the overall autophagy flux in cells [39]. Our results, shown in **Figures 3C and D**, demonstrated a decreased autophagic flux in the cells expressing QSOX1. Briefly, the LC3-II ratio was lower in MCF-7 QSOX1S-1 and QSOX1S-2 cells (2.8 and 1.4, respectively) compared to MCF-7 C cells (6.5) ***(*** ***Figure 3C*** ***)***. Similarly, this ratio was higher in MDA-MB-231 shQSOX1-2 and shQSOX1-1 cells (10.6 and 9.5, respectively) compared to MDA-MB-231 shC cells (6.3) ***(*** ***Figure 3D*** ***)***. Moreover, the p62 ratio was also decreased in the cells expressing QSOX1. This ratio was lower in MCF-7 QSOX1S-1 and QSOX1S-2 cells (1.6) compared to MCF-7 C cells (2.7) ***(*** ***Figure 3C*** ***)*** and higher in MDA-MB-231 shQSOX1-2 and shQSOX1-1 cells (3.3 and 2.4, respectively) compared to MDA-MB-231 shC cells (1.6) ***(*** ***Figure 3D*** ***)*** *.* These results prove that QSOX1 can inhibit the later stages of autophagy.

We then quantified the number of autophagosomes by studying GFP-LC3 fluorescence in our models in the presence or absence of bafilomycin A1. The results, described in the ***Figures 3E, F, G, H***, were consistent with those obtained for LC3-II levels in the western blotting experiments. Indeed, overexpression of QSOX1 in MCF-7 QSOX1S-1 and QSOX1S-2 cells enhanced the number of GFP-LC3 puncta per cell compared to MCF-7 C cells (23.7 and 20.4 vs 12.4) ***(*** ***Figures 3E and G*** ***)***. Likewise, knock-down of QSOX1 in MDA-MB-231 shQSOX1-2 and shQSOX1-1 cells led to a decrease of GFP-LC3 puncta per cell compared to MDA-MB-231 shC cells (9 and 8.4 vs 15.6) ***(*** ***Figures 3F and H*** ***)***. Moreover, treatment with bafilomycin A1 showed none or a limited effect on the cells expressing QSOX1. In fact, bafilomycin A1 enhanced the number of GFP-LC3 positive vesicles in MCF-7 C cells to those observed in MCF-7 QSOX1S-1 and QSOX1S-2 cells whereas this compound did not induce a further accumulation in these models. Similarly, bafilomycin A1 increased GFP-LC3 puncta in MDA-MB-231 shQSOX1-2 and shQSOX1-1 cells compared to MDA-MB-231 shC cells. These results confirm that QSOX1, like bafilomycin A1, can inhibit the later stages of autophagy (autophagosome/lysosome fusion or degradation by lysosomal proteases).

### QSOX1 Inhibits the Autophagosome/Lysosome Fusion

**Figure 4:**
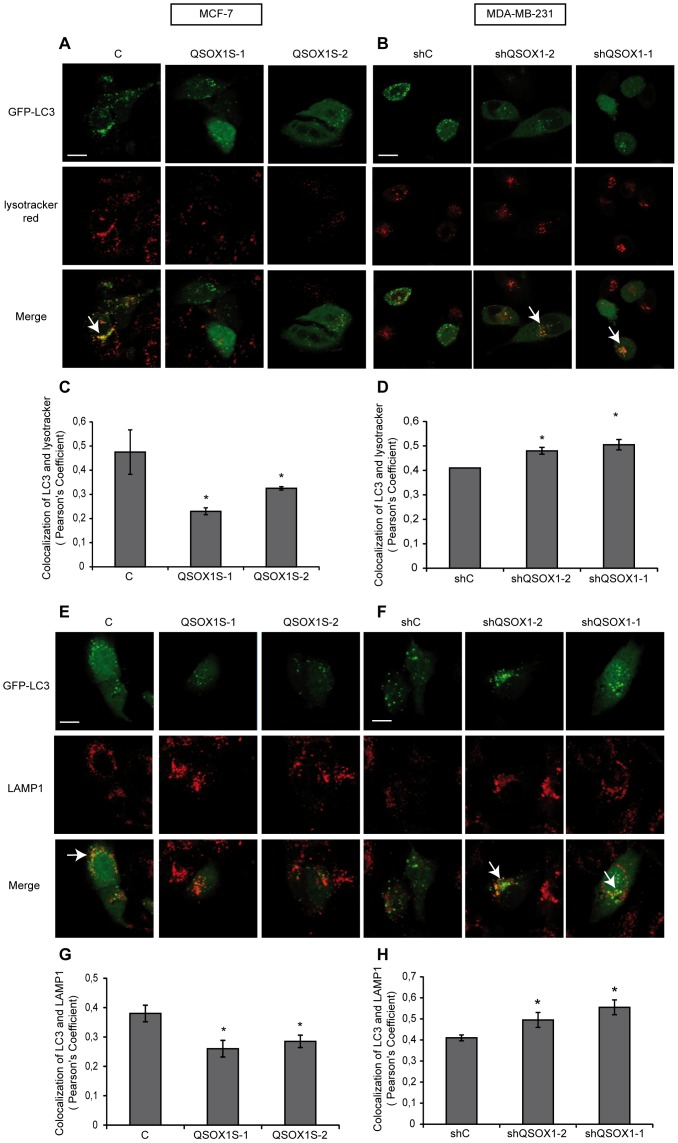
QSOX1 inhibits autophagosome/lysosome fusion. (**A**) MCF-7 C, QSOX1S-1, QSOX1S-2 and (**B**) MDA-MB-231 shC, shQSOX1-1, shQSOX1-2 cells were transfected with the pGFP-LC3 vector. 24 h after transfection, cells were incubated in complete medium supplemented with 500 nM Lysotracker red for 1 h. Scale bar represents 10 µm. (**C and D**) Colocalization between Lysotracker-stained acidic vesicles and GFP-LC3-positive autophagosomes, observed in A and B respectively, was quantified using a confocal microscope and the Pearson’s coefficient using coloc_2 plugin (ImageJ software). The data representative of two independent experiments are shown. *P<0.05 compared to the control. Arrows indicate colocalization. (**E**) MCF-7 C, QSOX1S-1, QSOX1S-2 and (**F**) MDA-MB-231 shC, shQSOX1-1, shQSOX1-2 cells were transfected with the pGFP-LC3 vector and then immunostained for LAMP1. Arrows indicate colocalization and Scale bar represents 10 µm. (**G and H**) Colocalization of the autophagosome marker GFP-LC3 and the lysosomal marker LAMP1 was analyzed using a confocal microscope and the Pearson’s coefficient using coloc_2 (ImageJ software). A representative image of two independent experiments is shown. *P<0.05 compared to the control.

We next investigated which stage of autophagy was targeted by QSOX1. Functional lysosomes are critical for the maturation of autophagosomes and the degradation of their content. We first examined the effect of QSOX1 on lysosomal acidification using the Lysotracker green marker, a marker of acidic compartments (including lysosomes) in living cells. Our data showed that there is no significant difference in Lysotracker staining in our different cell lines, suggesting that QSOX1 does not affect lysosomal function (Data not shown). To study autophagosome and lysosome fusion, we next examined the colocalization between Lysotracker red and GFP-LC3 fluorescence. Overexpression of QSOX1 in MCF-7 QSOX1S-1 and QSOX1S-2 cells led to a decrease of Lysotracker red/GFP-LC3 colocalization compared to MCF-7 C cells ***(*** ***Figures 4A and C*** ***)***. On the other hand, a knock-down of QSOX1 in MDA-MB-231 shQSOX1-2 and shQSOX1-1 cells led to an increase of Lysotracker red/GFP-LC3 colocalization compared to the one observed in MDA-MB-231 shC cells ***(*** ***Figures 4B and D*** ***)***. These results confirm that QSOX1 inhibits autophagosome/lysosome fusion. In order to further study the lysosome, we determined the GFP-LC3 colocalization with the lysosomal marker LAMP1. Our data showed that GFP-LC3 colocalized to a lesser extent with LAMP1 in the cells expressing QSOX1 compared to the cells that did not express this protein. Indeed, overexpression of QSOX1 in MCF-7 QSOX1S-1 and QSOX1S-2 cells led to a decrease of the LAMP1/GFP-LC3 colocalization ***(*** ***Figures 4E and G*** ***)*** and the knock-down of QSOX1 in MDA-MB-231 shQSOX1-2 and shQSOX1-1 cells led to an increase of LAMP1/GFP-LC3 colocalization ***(*** ***Figures 4F and H*** ***)***. Thus, these data confirm that QSOX1 inhibits autophagosome/lysosome fusion.

### QSOX1 Function in Cell Invasion is Related to its Role in Autophagy

**Figure 5:**
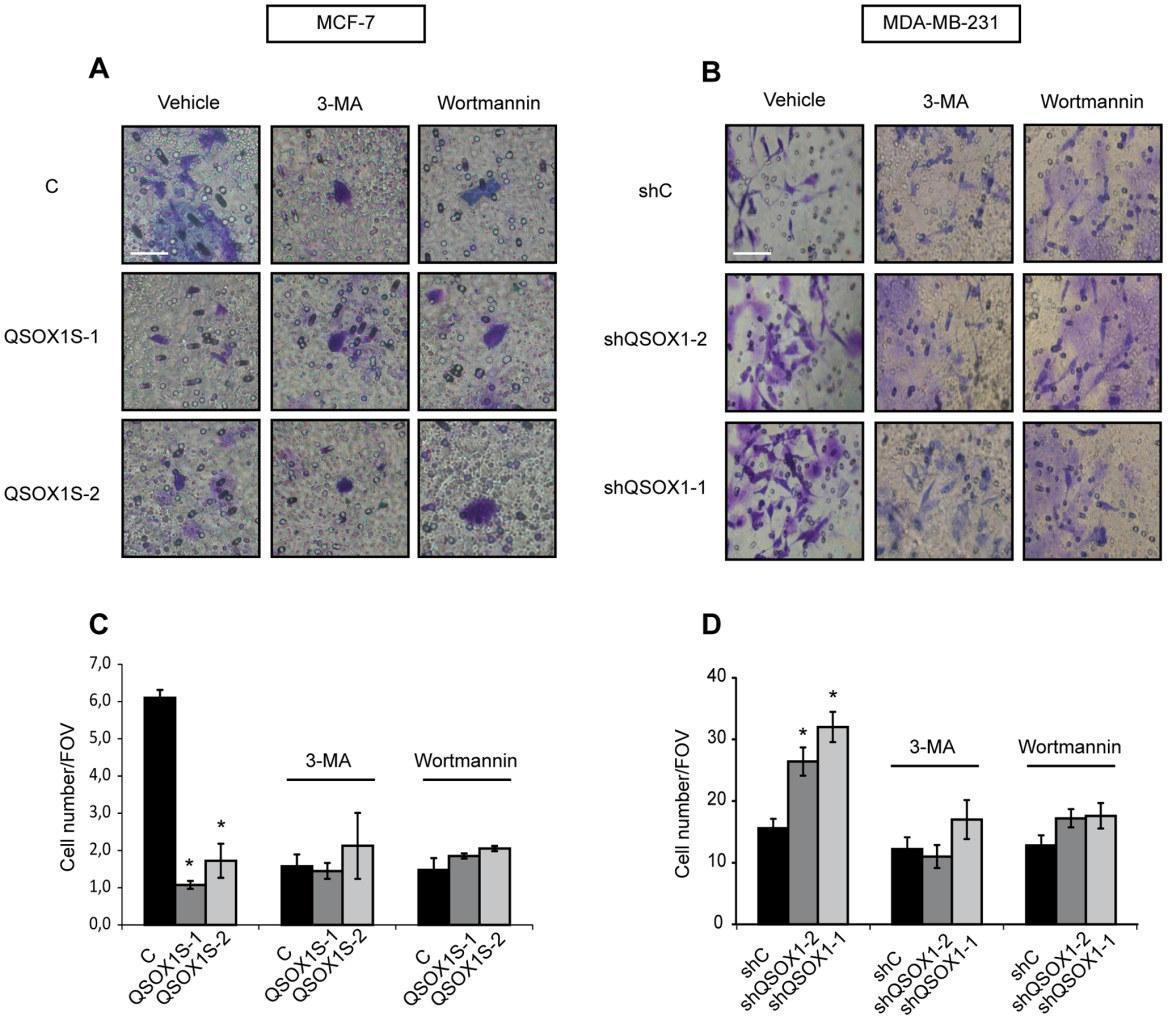
QSOX1 function in cell invasion is related to its role in autophagy. (**A**) MCF-7 C, QSOX1S-1, QSOX1S-2 and (**B**) MDA-MB-231 shC, shQSOX1-1, shQSOX1-2 cells were seeded on polycarbonate filters coated with Matrigel and incubated for 24 h, in the presence or absence of autophagy inhibitors 3-MA (10 mM) or wortmannin (100 nM). Inserts were then stained with a 2% crystal violet solution and photographed. A representative image of ten fields of view (FOV) of each membrane is shown. Scale bar represents 30 µm. (**C and D**) 10 FOV were randomly selected and the number of invasive cells, observed in A and B respectively, was determined. Data are means ± S.D. of two independent experiments performed in duplicate. *P<0.05 compared to the control.

We have previously shown in our laboratory that QSOX1 decreased breast cancer cell features such as invasion, clonogenicity and proliferation [11]. Moreover, several studies have shown that autophagy can promote cancer cell invasion [33], [40]. According to our results described above, which showed that QSOX1 inhibits autophagy, we hypothesized that this protein might inhibit invasion through its function on autophagy. To address this question, the effect of autophagy inhibitors on QSOX1 function in invasion assays was investigated. As previously described [11], overexpression of QSOX1 in MCF-7 QSOX1S-1 and QSOX1S-2 cells decreased cell invasion ***(*** ***Figures 5A and C*** ***)*** whereas knock-down of QSOX1 in MDA-MB-231 shQSOX1-2 and shQSOX1-1 cells increased this process ***(*** ***Figures 5B and D*** ***).*** After the inhibition of autophagy, using 3-MA or wortmannin, we no longer detected a difference in invasive abilities between the different cell lines expressing or not QSOX1. Indeed, the invasion ability of MCF-7 C cells was decreased to the levels observed in MCF-7 QSOX1S-1 and QSOX1S-2 cells ***(*** ***Figures 5A and C*** ***)*** **.** Similarly, invasion ability of MDA-MB-231 shQSOX1-2 and QSOX1-1 cells were decreased to the levels observed in MDA-MB-231 shC cells ***(*** ***Figures 5B and D*** ***)***. Therefore, we can conclude that the inhibition of autophagy by 3-MA or wortmannin treatment mimics the effect of QSOX1 on cell invasion. These results suggest that QSOX1 might inhibit cancer cell invasion via its function in autophagy.

### The Extinction of QSOX1 Expression in Tumors Decreases p62 Levels

**Figure 6:**
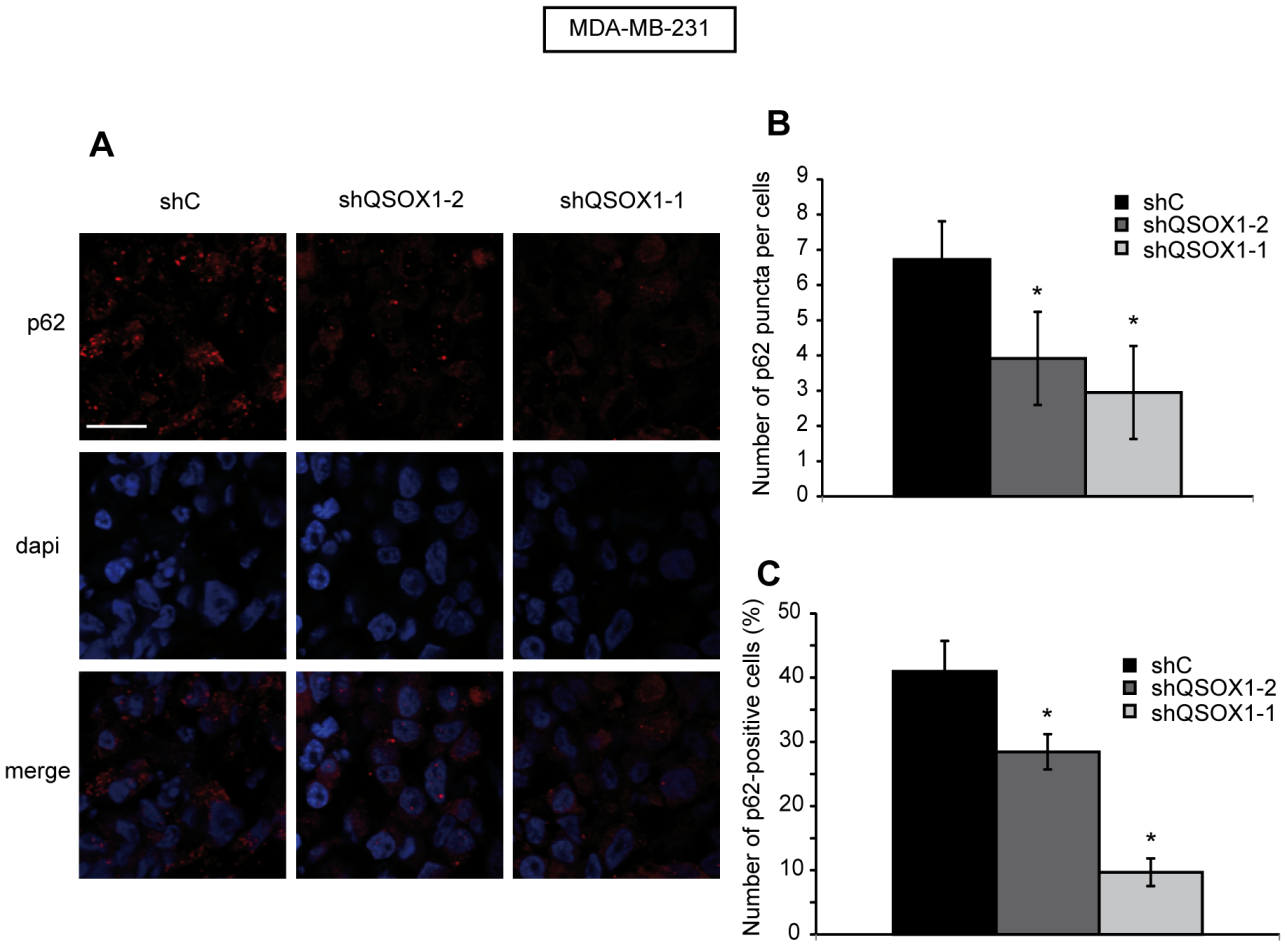
The extinction of QSOX1 expression in tumors is correlated with low levels of p62. (**A**) Tissue sections of MDA-MB-231 shC, shQSOX1-2 and shQSOX1-1 tumors fixed in formol were subjected to p62 immunostaining. Sections were then analyzed by confocal microscopy and a representative image of 3 independent experiments performed in duplicate is shown. Scale bar represents 30 µm. (**B**) The number of p62 puncta per cell and (**C**) the number of p62-positive cells were determined using the ImageJ software. To determine the number of p62 puncta, 40 cells per tumor were randomly counted. To determine the number of p62-positive cells count, 23 fields were randomly chosen. *P<0.05 compared to the control.

We have previously shown that a decreased expression of QSOX1 in subcutaneous mouse xenografts led to a strong enhancement of the tumor growth. Indeed, tumors obtained after inoculation of MDA-MB-231 shQSOX1-1 cells presented a higher volume than those obtained after inoculation of MDA-MB-231 shC cells [11]. Similar results were obtained after inoculation of MDA-MB-231 shQSOX1-2 cells ***(Figure S1).*** In order to investigate the effect of QSOX1 on autophagy *in vivo*, we studied whether autophagic marker levels such as LC3 and p62 were altered in these tumors. No significant differences were observed for LC3 levels (data not shown) but, as shown in ***Figures 6A, B and C***, MDA-MB-231 shC tumors exhibited higher levels of p62 puncta per cell and a higher number of p62-positive cells compared to the levels observed in the MDA-MB-231 shQSOX1-2 and shQSOX1-1 tumors. Altogether, these data demonstrate that QSOX1 extinction activates p62 degradation in breast tumors. These results are in agreement with those described above at the cellular level and suggest that QSOX1 function in tumor growth *in vivo* could be linked to its inhibiting effect on autophagy.

## Discussion

In this study, we demonstrate for the first time that QSOX1 plays a role in autophagy through the inhibition of autophagosome/lysosome fusion in breast cancer cells.

While the enzymatic function of QSOX1 is well described, its biological functions are not fully elucidated. QSOX1 expression is deregulated in cancer cells and this protein has been described to be involved in tumorigenesis. We previously demonstrated that QSOX1 is induced following an oxidative [15] or ER stress (unpublished data), and protects breast cancer cells against a stress-induced cell death. Since autophagy is a stress-regulated mechanism involved in cancer progression, we investigated whether QSOX1 could regulate the autophagic pathway, a regulation that could explain its role during tumor development. To do so, we used two models of breast cancer cell lines: a MCF-7 cell line in which QSOX1 is overexpressed and a MDA-MB-231 cell line in which QSOX1 is extinguished.

During a nutrient stress-induced autophagy, QSOX1 mRNA and protein levels were upregulated and cell survival increased, as it has already been shown for other cellular stresses. Besides its role in homeostasis and cell survival, autophagy can be associated to a non-apoptotic form of cell death called autophagic cell death or type II programmed cell death. Indeed, this type of cell death can occur when autophagy is excessive or when the cells fail to survive against cellular stresses [41], [42], [43], [44]. As such, during an extended amino acid starvation, QSOX1 might prevent excessive autophagy and/or autophagic cell death and allow cell survival by inhibiting this catabolic process. As such, it would be interesting to focus future experiments on the study of autophagic cell death in our different cell models.

Since QSOX1 is upregulated during autophagy induction, its role in this process was investigated through the study of different autophagic markers. The results regarding p62 degradation suggest that QSOX1 inhibits the basal autophagic flux. The increase in LC3-II protein levels could have represented either an increase of autophagy induction or a decrease of autophagosome degradation. To clarify this point, we compared the ratio of LC3-II in the presence and the absence of the lysosomal inhibitor bafilomycin A1. Our results showed that QSOX1 overexpression leads to an increase in p62 and LC3-II levels and an increase in the number of GFP-LC3 puncta in breast cancer cells. More interestingly, bafilomycin A1, which is an autophagosome/lysosome fusion inhibitor, had a limited effect on autophagic flux in cells overexpressing QSOX1. We also observed that QSOX1 prevents the colocalization of GFP-LC3 (an autophagosome marker) and Lysotracker (a marker of acidic vesicles) or LAMP1 (a lysosomal marker) staining, suggesting that this protein inhibits the autophagosome/lysosome fusion. These results are consistent with those obtained in a genome-wide human siRNA screen targeting more than 20 000 genes, suggesting that the knock-down of QSOX1 leads to an increase of GFP-LC3 puncta in the absence, but not in the presence of a lysosomal protease inhibitor (E64d) in neuroblastoma cells [45].

The mechanism by which QSOX1 inhibits autophagy remains to be determined. One of the potential QSOX1 interacting proteins is Ubiquilin4 (UBQLN4), a protein which belongs to the Ubiquilin family involved in the autophagic process [46]. Indeed, it has recently been shown that UBQLN4 acts together with UBQLN1 to induce autophagosome/lysosome fusion through mediated interaction of UBQNL1 with the autophagic machinery [47], [48]. QSOX1, through its interaction with UBQLN4, could inhibit the autophagosome/lysosome fusion. Moreover, a genome-wide targeting more than 200 genes has shown that QSOX1 (QSCN6) could inhibit autophagy in human neuroblastoma cells by negatively regulating PI3K activity [49]. These results suggest that QSOX1 could inhibit autophagosome/lysosome fusion by interfering with the formation of the complex between UV radiation resistance-associated gene (UVRAG), phosphoinositide 3-kinase (PI3K) and Beclin1.

Several previous studies have shown an involvement of QSOX1 in cancer. However, the role of QSOX1 seems to be complex mainly because of the existence of its different transcripts. Indeed, expression of QSOX1 spliced variants present differential expression patterns depending on the type of cancer and the specific role of these different transcripts remain elusive. Consequently, QSOX1 has been correlated to a good or a poor prognosis according to the type of cancer studied and could promote or reduce cancer cell phenotypes according to the tumor type or grade [1], [11], [12], [13], [14], [15], [16], [17], [18].

Autophagy also plays a role in various cancers such as breast cancer. This process has a “double-edged sword” role in cancer depending on the cell type, the context or the stage of tumor development [50], [51], [52]. As such, we wondered whether the involvement of QSOX1 in autophagy might explain its function in tumor progression. Our results suggest that QSOX1, through the inhibition of autophagy, reduces cell invasion. To confirm this hypothesis, it will be necessary to genetically inhibit autophagy using siRNAs against ATG5 or ATG7 and study the effect of this inhibition on QSOX1 function in invasion. These results also highlight that autophagy plays a prosurvival role in our cellular models as previously described in pancreatic or mammary tumorigenesis [53], [54], [55].

It has previously been shown in our laboratory that QSOX1 inhibits tumor growth *in vivo* [11]. Indeed, tumors obtained after inoculation of MDA-MB-231 shQSOX1 cells in NOG mice presented a higher volume than those obtained after inoculation of MDA-MB-231 shC cells. In order to determine whether these differences could be related to a deregulation of autophagy, we investigated the expression of autophagic markers (p62 and LC3-II) in these tumors. Our data showed no significant differences for LC3 levels between shC and shQSOX1 tumors (Data not shown) but the presence of QSOX1 in control tumors was associated with high levels of p62. These results are in agreement with those observed in our cellular models and suggest that QSOX1 plays a role during tumor growth *in vivo* and that this effect could be linked to its inhibitory effect on autophagy. However, tumors-induced by MDA-MB-231 shQSOX1-2 cells, which present 92% of QSOX1 extinction, did not present a greater decrease of p62 levels compared to tumors-induced by MDA-MB-231 shQSOX1-1 cells which present 55% of QSOX1 extinction, as expected in regard to the results obtained *in vitro* with the two cell lines. Previous results have already demonstrated that tumors obtained after inoculation of MDA-MB-231 shQSOX1-1 [11] and shQSOX1-2 (Figure S1) cells show similar growth despite their difference in the level of QSOX1 extinction. This observation could be explained by the fact that a tumor tissue is far more complex and heterogeneous than a cell line *in vitro*. Moreover, p62 is a multi-domain protein that interacts with the autophagic machinery and plays a role in various signaling pathways such as antioxidant response, inflammation, metabolism and cancer [56], we still cannot exclude that the high p62 levels observed in tumors expressing QSOX1 might not only be related to the deregulation of autophagy. Moreover, our results are consistent with those previously reported describing a pro-survival role of autophagy during tumor establishment and progression [33], [57].

**Figure 7:**
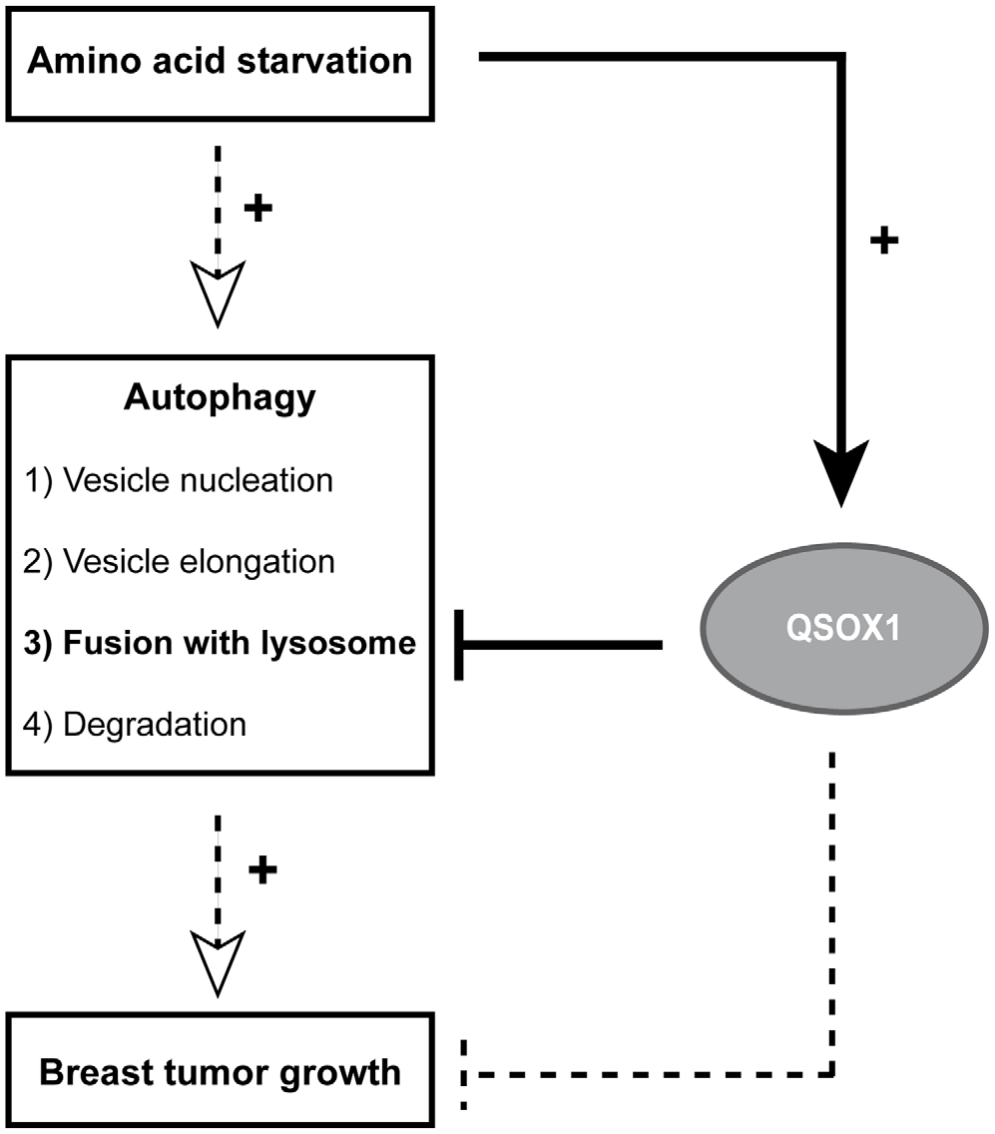
QSOX1 functions in breast cancer cells. Our results demonstrate that QSOX1 expression is induced following a nutrient stress-induced autophagy. QSOX1 also inhibits autophagy through the inhibition of autophagosome/lysosome fusion and its inhibitory effect on autophagy could explain its function in breast cancer cell invasion and tumor growth. Dotted arrows and lines represent data which have already been described whereas solid arrows and lines represent results obtained in our study.

Altogether, our results demonstrate that QSOX1 inhibits the autophagic pathway through the inhibition of autophagosome/lysosome fusion and that its inhibitory effect on autophagy might explain its function in cancer cell invasion and tumor growth (Figure 7).

## Supporting Information

Figure S1 **Decreased expression of QSOX1 led to a strong increase in tumor growth.** MDA-MB-231 shC, shQSOX1-1 and shQSOX1-2 cells were injected subcutaneously in CIEA NOG mice (n = 5 per group). **(A)** 21 days after injection, the evolution of the tumor volume was measured twice a week. The tumor volume was calculated using the formula: V = ½ a × b2, where a is the longest tumor axis, and b is the shortest tumor axis. **(B)** 42 days after injection, tumors were fixed in formol and photographed. * P<0.05, compared to the control. This experiment is representative of two independent experiments.
(TIF)

## References

[1] Musard JF, Sallot M, Dulieu P, Fraichard A, Ordener C, et al (2001) Identification and expression of a new sulfhydryl oxidase SOx-3 during the cell cycle and the estrus cycle in uterine cells. Biochem Biophys Res Commun 287: 83–91. 11549257 10.1006/bbrc.2001.5440

[2] Thorpe C, Hoober KL, Raje S, Glynn NM, Burnside J, et al (2002) Sulfhydryl oxidases: emerging catalysts of protein disulfide bond formation in eukaryotes. Arch Biochem Biophys 405: 1–12. 12176051 10.1016/s0003-9861(02)00337-5

[3] Heckler EJ, Rancy PC, Kodali VK, Thorpe C (2008) Generating disulfides with the Quiescin-sulfhydryl oxidases. Biochim Biophys Acta 1783: 567–577. 17980160 10.1016/j.bbamcr.2007.10.002 PMC2374921

[4] Tury A, Mairet-Coello G, Poncet F, Jacquemard C, Risold PY, et al (2004) QSOX sulfhydryl oxidase in rat adenohypophysis: localization and regulation by estrogens. J Endocrinol 183: 353–363. 15531723 10.1677/joe.1.05842

[5] Benayoun B, Esnard-Feve A, Castella S, Courty Y, Esnard F (2001) Rat seminal vesicle FAD-dependent sulfhydryl oxidase. Biochemical characterization and molecular cloning of a member of the new sulfhydryl oxidase/quiescin Q6 gene family. J Biol Chem 276: 13830–13837. 11278790 10.1074/jbc.M010933200

[6] Hoober KL, Joneja B, White HB 3rd, Thorpe C (1996) A sulfhydryl oxidase from chicken egg white. J Biol Chem 271: 30510–30516. 8940019 10.1074/jbc.271.48.30510

[7] Ilani T, Alon A, Grossman I, Horowitz B, Kartvelishvily E, et al (2013) A secreted disulfide catalyst controls extracellular matrix composition and function. Science 341: 74–76. 23704371 10.1126/science.1238279

[8] Song H, Zhang B, Watson MA, Humphrey PA, Lim H, et al (2009) Loss of Nkx3.1 leads to the activation of discrete downstream target genes during prostate tumorigenesis. Oncogene 28: 3307–3319. 19597465 10.1038/onc.2009.181 PMC2746257

[9] Antwi K, Hostetter G, Demeure MJ, Katchman BA, Decker GA, et al (2009) Analysis of the plasma peptidome from pancreas cancer patients connects a peptide in plasma to overexpression of the parent protein in tumors. J Proteome Res 8: 4722–4731. 19795908 10.1021/pr900414f

[10] Hellebrekers DM, Castermans K, Vire E, Dings RP, Hoebers NT, et al (2006) Epigenetic regulation of tumor endothelial cell anergy: silencing of intercellular adhesion molecule-1 by histone modifications. Cancer Res 66: 10770–10777. 17108113 10.1158/0008-5472.CAN-06-1609

[11] Pernodet N, Hermetet F, Adami P, Vejux A, Descotes F, et al (2012) High expression of QSOX1 reduces tumorogenesis, and is associated with a better outcome for breast cancer patients. Breast Cancer Res 14: R136. 23098186 10.1186/bcr3341 PMC4053115

[12] Hellebrekers DM, Melotte V, Vire E, Langenkamp E, Molema G, et al (2007) Identification of epigenetically silenced genes in tumor endothelial cells. Cancer Res 67: 4138–4148. 17483324 10.1158/0008-5472.CAN-06-3032

[13] Coppock DL, Kopman C, Scandalis S, Gilleran S (1993) Preferential gene expression in quiescent human lung fibroblasts. Cell Growth Differ 4: 483–493. 8396966

[14] Coppock D, Kopman C, Gudas J, Cina-Poppe DA (2000) Regulation of the quiescence-induced genes: quiescin Q6, decorin, and ribosomal protein S29. Biochem Biophys Res Commun 269: 604–610. 10708601 10.1006/bbrc.2000.2324

[15] Morel C, Adami P, Musard JF, Duval D, Radom J, et al (2007) Involvement of sulfhydryl oxidase QSOX1 in the protection of cells against oxidative stress-induced apoptosis. Exp Cell Res 313: 3971–3982. 17927979 10.1016/j.yexcr.2007.09.003

[16] Katchman BA, Antwi K, Hostetter G, Demeure MJ, Watanabe A, et al (2011) Quiescin sulfhydryl oxidase 1 promotes invasion of pancreatic tumor cells mediated by matrix metalloproteinases. Mol Cancer Res 9: 1621–1631. 21989104 10.1158/1541-7786.MCR-11-0018

[17] Katchman BA, Ocal IT, Cunliffe HE, Chang YH, Hostetter G, et al (2013) Expression of quiescin sulfhydryl oxidase 1 is associated with a highly invasive phenotype and correlates with a poor prognosis in Luminal B breast cancer. Breast Cancer Res 15: R28. 23536962 10.1186/bcr3407 PMC3738157

[18] Soloviev M, Esteves MP, Amiri F, Crompton MR, Rider CC (2013) Elevated transcription of the gene QSOX1 encoding quiescin Q6 sulfhydryl oxidase 1 in breast cancer. PLoS One 8: e57327. 23460839 10.1371/journal.pone.0057327 PMC3583868

[19] Galluzzi L, Vicencio JM, Kepp O, Tasdemir E, Maiuri MC, et al (2008) To die or not to die: that is the autophagic question. Curr Mol Med 8: 78–91. 18336289 10.2174/156652408783769616

[20] Cuervo AM (2004) Autophagy: in sickness and in health. Trends Cell Biol 14: 70–77. 15102438 10.1016/j.tcb.2003.12.002

[21] Xie Z, Klionsky DJ (2007) Autophagosome formation: core machinery and adaptations. Nat Cell Biol 9: 1102–1109. 17909521 10.1038/ncb1007-1102

[22] Zhou S, Zhao L, Kuang M, Zhang B, Liang Z, et al (2012) Autophagy in tumorigenesis and cancer therapy: Dr. Jekyll or Mr. Hyde? Cancer Lett 323: 115–127. 22542808 10.1016/j.canlet.2012.02.017

[23] Liu EY, Ryan KM (2012) Autophagy and cancer–issues we need to digest. J Cell Sci 125: 2349–2358. 22641689 10.1242/jcs.093708

[24] Lozy F, Karantza V (2012) Autophagy and cancer cell metabolism. Semin Cell Dev Biol 23: 395–401. 22281437 10.1016/j.semcdb.2012.01.005 PMC3639127

[25] Mathew R, Kongara S, Beaudoin B, Karp CM, Bray K, et al (2007) Autophagy suppresses tumor progression by limiting chromosomal instability. Genes Dev 21: 1367–1381. 17510285 10.1101/gad.1545107 PMC1877749

[26] Karantza-Wadsworth V, Patel S, Kravchuk O, Chen G, Mathew R, et al (2007) Autophagy mitigates metabolic stress and genome damage in mammary tumorigenesis. Genes Dev 21: 1621–1635. 17606641 10.1101/gad.1565707 PMC1899472

[27] Liang XH, Jackson S, Seaman M, Brown K, Kempkes B, et al (1999) Induction of autophagy and inhibition of tumorigenesis by beclin 1. Nature 402: 672–676. 10604474 10.1038/45257

[28] Liang C, Feng P, Ku B, Dotan I, Canaani D, et al (2006) Autophagic and tumour suppressor activity of a novel Beclin1-binding protein UVRAG. Nat Cell Biol 8: 688–699. 16799551 10.1038/ncb1426

[29] Kang MR, Kim MS, Oh JE, Kim YR, Song SY, et al (2009) Frameshift mutations of autophagy-related genes ATG2B, ATG5, ATG9B and ATG12 in gastric and colorectal cancers with microsatellite instability. J Pathol 217: 702–706. 19197948 10.1002/path.2509

[30] Aita VM, Liang XH, Murty VV, Pincus DL, Yu W, et al (1999) Cloning and genomic organization of beclin 1, a candidate tumor suppressor gene on chromosome 17q21. Genomics 59: 59–65. 10395800 10.1006/geno.1999.5851

[31] Harris AL (2002) Hypoxia–a key regulatory factor in tumour growth. Nat Rev Cancer 2: 38–47. 11902584 10.1038/nrc704

[32] Brahimi-Horn MC, Bellot G, Pouyssegur J (2011) Hypoxia and energetic tumour metabolism. Curr Opin Genet Dev 21: 67–72. 21074987 10.1016/j.gde.2010.10.006

[33] Wei H, Wei S, Gan B, Peng X, Zou W, et al (2011) Suppression of autophagy by FIP200 deletion inhibits mammary tumorigenesis. Genes Dev 25: 1510–1527. 21764854 10.1101/gad.2051011 PMC3143941

[34] Haan C, Behrmann I (2007) A cost effective non-commercial ECL-solution for Western blot detections yielding strong signals and low background. J Immunol Methods 318: 11–19. 17141265 10.1016/j.jim.2006.07.027

[35] Jain A, Lamark T, Sjottem E, Larsen KB, Awuh JA, et al (2010) p62/SQSTM1 is a target gene for transcription factor NRF2 and creates a positive feedback loop by inducing antioxidant response element-driven gene transcription. J Biol Chem 285: 22576–22591. 20452972 10.1074/jbc.M110.118976 PMC2903417

[36] Duran A, Linares JF, Galvez AS, Wikenheiser K, Flores JM, et al (2008) The signaling adaptor p62 is an important NF-kappaB mediator in tumorigenesis. Cancer Cell 13: 343–354. 18394557 10.1016/j.ccr.2008.02.001

[37] Puissant A, Auberger P (2010) AMPK- and p62/SQSTM1-dependent autophagy mediate resveratrol-induced cell death in chronic myelogenous leukemia. Autophagy 6: 655–657. 20458181 10.4161/auto.6.5.12126

[38] Klionsky DJ, Abdalla FC, Abeliovich H, Abraham RT, Acevedo-Arozena A, et al (2012) Guidelines for the use and interpretation of assays for monitoring autophagy. Autophagy 8: 445–544. 22966490 10.4161/auto.19496 PMC3404883

[39] Arduino DM, Esteves AR, Cortes L, Silva DF, Patel B, et al (2012) Mitochondrial metabolism in Parkinson’s disease impairs quality control autophagy by hampering microtubule-dependent traffic. Hum Mol Genet 21: 4680–4702. 22843496 10.1093/hmg/dds309 PMC3471400

[40] Macintosh RL, Timpson P, Thorburn J, Anderson KI, Thorburn A, et al (2012) Inhibition of autophagy impairs tumor cell invasion in an organotypic model. Cell Cycle 11: 2022–2029. 22580450 10.4161/cc.20424 PMC3359125

[41] Dalby KN, Tekedereli I, Lopez-Berestein G, Ozpolat B (2010) Targeting the prodeath and prosurvival functions of autophagy as novel therapeutic strategies in cancer. Autophagy 6: 322–329. 20224296 10.4161/auto.6.3.11625 PMC2914492

[42] Clarke R, Cook KL, Hu R, Facey CO, Tavassoly I, et al (2012) Endoplasmic reticulum stress, the unfolded protein response, autophagy, and the integrated regulation of breast cancer cell fate. Cancer Res 72: 1321–1331. 22422988 10.1158/0008-5472.CAN-11-3213 PMC3313080

[43] Levine B, Yuan J (2005) Autophagy in cell death: an innocent convict? J Clin Invest 115: 2679–2688. 16200202 10.1172/JCI26390 PMC1236698

[44] Kroemer G, Levine B (2008) Autophagic cell death: the story of a misnomer. Nat Rev Mol Cell Biol 9: 1004–1010. 18971948 10.1038/nrm2527 PMC2727358

[45] Lipinski MM, Hoffman G, Ng A, Zhou W, Py BF, et al (2010) A genome-wide siRNA screen reveals multiple mTORC1 independent signaling pathways regulating autophagy under normal nutritional conditions. Dev Cell 18: 1041–1052. 20627085 10.1016/j.devcel.2010.05.005 PMC2935848

[46] Lim J, Hao T, Shaw C, Patel AJ, Szabo G, et al (2006) A protein-protein interaction network for human inherited ataxias and disorders of Purkinje cell degeneration. Cell 125: 801–814. 16713569 10.1016/j.cell.2006.03.032

[47] N’Diaye EN, Debnath J, Brown EJ (2009) Ubiquilins accelerate autophagosome maturation and promote cell survival during nutrient starvation. Autophagy 5: 573–575. 19398896 10.4161/auto.5.4.8312

[48] Yun Lee D, Arnott D, Brown EJ (2013) Ubiquilin4 is an adaptor protein that recruits Ubiquilin1 to the autophagy machinery. EMBO Rep 14: 373–381. 23459205 10.1038/embor.2013.22 PMC3615663

[49] Lipinski MM, Zheng B, Lu T, Yan Z, Py BF, et al (2010) Genome-wide analysis reveals mechanisms modulating autophagy in normal brain aging and in Alzheimer’s disease. Proc Natl Acad Sci U S A 107: 14164–14169. 20660724 10.1073/pnas.1009485107 PMC2922576

[50] Berardi DE, Campodonico PB, Diaz Bessone MI, Urtreger AJ, Todaro LB (2011) Autophagy: friend or foe in breast cancer development, progression, and treatment. Int J Breast Cancer 2011: 595092. 22295229 10.4061/2011/595092 PMC3262577

[51] Debnath J (2011) The multifaceted roles of autophagy in tumors-implications for breast cancer. J Mammary Gland Biol Neoplasia 16: 173–187. 21779879 10.1007/s10911-011-9223-3 PMC3170851

[52] Karantza-Wadsworth V, White E (2007) Role of autophagy in breast cancer. Autophagy 3: 610–613. 17786023 10.4161/auto.4867 PMC2859167

[53] Gong C, Bauvy C, Tonelli G, Yue W, Delomenie C, et al (2013) Beclin 1 and autophagy are required for the tumorigenicity of breast cancer stem-like/progenitor cells. Oncogene 32: 2261–2272. 22733132 10.1038/onc.2012.252 PMC3679409

[54] Yang S, Wang X, Contino G, Liesa M, Sahin E, et al (2011) Pancreatic cancers require autophagy for tumor growth. Genes Dev 25: 717–729. 21406549 10.1101/gad.2016111 PMC3070934

[55] Amaravadi RK (2012) Autophagy and tumor cell invasion. Cell Cycle 11: 3718–3719. 22982997 10.4161/cc.22147 PMC3495808

[56] Puissant A, Fenouille N, Auberger P (2012) When autophagy meets cancer through p62/SQSTM1. Am J Cancer Res 2: 397–413. 22860231 PMC3410580

[57] Mathew R, Karantza-Wadsworth V, White E (2009) Assessing metabolic stress and autophagy status in epithelial tumors. Methods Enzymol 453: 53–81. 19216902 10.1016/S0076-6879(08)04004-4 PMC2857509

