# Supplementary figures and images for: QSOX1 Inhibits Autophagic Flux in Breast Cancer Cells
Source: PLoS One. 2014 Jan 24;9(1):e86641. doi: 10.1371/journal.pone.0086641 (PMC3901705; doi:10.1371/journal.pone.0086641)

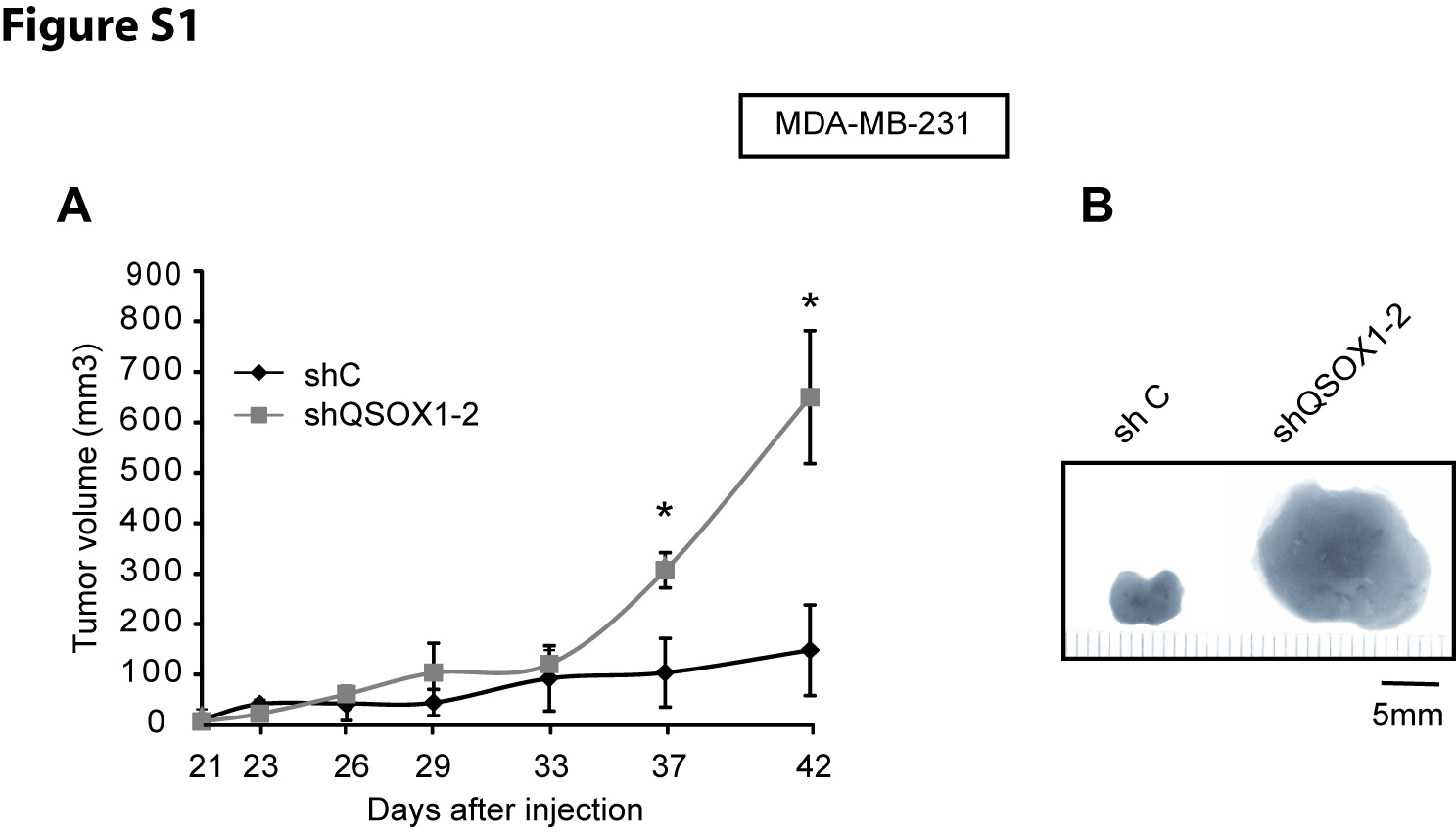

Supplement: Figure S1 — Decreased expression of QSOX1 led to a strong increase in tumor growth. MDA-MB-231 shC, shQSOX1-1 and shQSOX1-2 cells were injected subcutaneously in CIEA NOG mice (n = 5 per group). (A) 21 days after injection, the evolution of the tumor volume was measured twice a week. The tumor volume was calculated using the formula: V = ½ a × b2, where a is the longest tumor axis, and b is the shortest tumor axis. (B) 42 days after injection, tumors were fixed in formol and photographed. * P<0.05, compared to the control. This experiment is representative of two independent experiments. (TIF) [file pone.0086641.s001.tif]
